# Involuntary Facial Expression Processing: Extracting Information from Two Simultaneously Presented Faces

**DOI:** 10.1371/journal.pone.0022287

**Published:** 2011-07-22

**Authors:** Samantha Baggott, Romina Palermo, Mark A. Williams

**Affiliations:** 1 ARC Centre for Cognition and its Disorders and Macquarie Centre for Cognitive Science (MACCS), Macquarie University, Sydney, New South Wales, Australia; 2 ARC Centre for Cognition and its Disorders, Department of Psychology, The Australian National University, Canberra, Australian Capital Territory, Australia; University of London, United Kingdom

## Abstract

Facial expressions play an important role in successful social interactions, with previous research suggesting that facial expressions may be processed involuntarily. In the current study, we investigate whether involuntary processing of facial expressions would also occur when facial expression distractors are simultaneously presented in the same spatial location as facial expression targets. Targets and distractors from another stimulus class (lions) were also used. Results indicated that angry facial expression distractors interfered more than neutral face distractors with the ability to respond to both face and lion targets. These findings suggest that information from angry facial expressions can be extracted rapidly from a very brief presentation (50 ms), providing compelling evidence that angry facial expressions are processed involuntarily.

## Introduction

In everyday life we are constantly exposed to a vast number of competing sources of information. To simply drive along a busy street, for example, it is critical that we attend to relevant information (e.g., other vehicles, pedestrians, traffic lights and road signs) at the expense of irrelevant information that may also demand attention (e.g., flashing advertisements). It seems to be particularly difficult, though, to ignore evolutionarily or biologically specified irrelevant information. Faces are biologically and socially important for signalling the race, age and sex of an individual, as well as whether they are friend or foe. It follows, then, that irrelevant faces should be difficult to ignore. Consistent with this, recent studies have demonstrated that the sex and identity of a face continues to be processed even when that face is task irrelevant, suggesting that some aspects of face processing occur involuntarily [1]–[4].

Beyond the sex and identity information conveyed by faces, facial expressions also communicate important social information. Owing to the pivotal role facial expressions play in social interactions, facial expressions, too, ought to be difficult to ignore. By superimposing emotional word targets on facial expression distractors that were congruent, incongruent or neutral with respect to the emotional content of the word, previous studies have suggested that facial expressions continue to be processed even when task irrelevant ([5], [6]; see also [7], [8]). That is, responding to the emotional category of the word target was found to be faster when face distractors were emotionally congruent, as compared to when they were emotionally incongruent. Thus, as for sex and identity information, the processing of facial expressions has also been argued to occur involuntarily.

The evidence for the processing of irrelevant emotional facial expressions is based on the finding that incongruent *facial expression distractors* interfere with the ability to respond to *emotional word targets*. However, there are important differences between facial expressions and words in terms of their visual characteristics, their social and evolutionary significance, and the way in which they are represented and processed [9], [10]. Thus, the aim of the current study was to investigate whether involuntary facial expression processing would continue to be observed when target-distractor dissimilarity is reduced. An ideal way to do this involves pairing *face targets with face distractors*.

Few studies have used faces as both targets and distractors. A rare exception is the series of experiments conducted by Bindemann et al. [1], in which participants categorised central targets while ignoring congruent or incongruent flanking distractors. In these experiments, incongruent face distractors interfered with target names to be categorised as male or female, incongruent famous face distractors interfered with target famous names to be categorised as pop-stars or politicians, and incongruent famous face distractors interfered with target flags to be categorised as British or American. Regardless of the required judgement, though, no interference was found for face distractors paired with face targets, suggesting that it is only information from the target face that is processed. This finding is also consistent with a number of previous facial identity studies which have suggested that information from only the one face is processed at the one time (e.g., [11]–[14]).

Despite the suggestion from facial identity tasks that information cannot be processed from two simultaneously presented faces, there is evidence suggesting that the same may not be true for *facial expressions*. Results from several neuroimaging studies suggest that the amygdala, a region involved in facial expression perception, responds to facial expressions even when they are unattended ([15]–[18]; though see [19] for evidence that this may not occur under conditions of high attentional load). Given the evidence indicating that the amygdala responds even to unattended facial expressions, it might be expected that emotional information could be processed from both relevant and irrelevant facial expressions.

**Figure 1:**
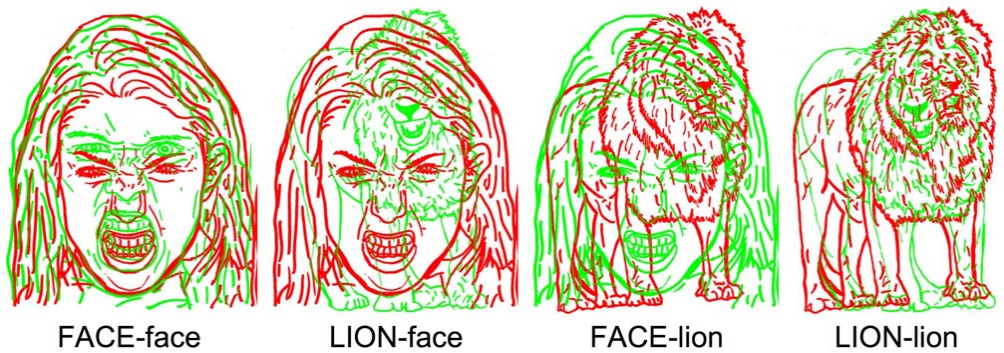
Examples of the composite displays for each of the target-distractor pairings. These composite displays depict female face images and male lion images, all displaying anger. The target image is green in this example. Faces were selected from the NimStim Face Stimulus Set (see [26]).

The condition of primary interest in the current study, then, concerned face targets being paired with face distractors. Additionally, targets and distractors were selected from a non-face stimulus class: profiles of lions (see Figure 1). Profiles of lions were chosen as they are more similar to faces in terms of size, spatial frequency, the way they are processed and represented within the visual system [9], [10], and in terms of biological and social relevance than the word targets used in previous experiments.

Targets and distractors (either faces or lions) were superimposed to ensure that the spatial location of the images was held constant (similar to previous studies with words: [5]–[8]). In contrast to other phenomena such as bistable perception or binocular rivalry (e.g., [17], [20], [21]), the superimposing of targets and distractors was also important for ensuring equivalence between the veridical and perceived stimuli. Targets were specified to participants on the basis of colour, a pre-attentive feature that can guide visual attention [22], [23]. The colour of target images was counterbalanced across participants.

We selected images of human faces and profiles of lions that were either angry or emotionally neutral to permit comparisons between the amount of interference from emotional and emotionally neutral distractors. Anger was chosen as the emotion of interest based on the anger-superiority hypothesis which suggests that angry facial expressions are subject to particularly efficient processing [24], [25]. Anger is also an evolutionary salient signal of threat, and the most likely emotion to be associated with the lion images used as the non-face stimulus class.

Male and female faces and lions and lionesses were used, and participants were asked to categorise the sex of the targets. Importantly, we used a sex categorisation task to investigate whether interference from irrelevant facial expressions would be present when the emotional manipulation was completely unconnected to the classification required of the target.

To summarise, the current study aimed to test the hypothesis that facial expressions are involuntarily processed, without the difficulties associated with having dissimilar targets and distractors (i.e., faces and words) and when emotion is completely unrelated to the demands of the task.

As noted, the condition in which face targets were paired with face distractors was of primary interest. Given results suggesting that facial expression distractors influence responding to non-face *word* targets, we anticipate that angry facial expression distractors would interfere more than neutral facial expression distractors when judging the sex of non-face (*lion*) targets. This result would indicate that task irrelevant angry facial expressions are processed, even when the non-face (lion) targets are more similar to faces than the previously used non-face (word) targets.

If information from only one facial expression can be processed at a time, then we would not expect a difference between angry and neutral facial expression distractors when judging the sex of *face* targets. If, by contrast, angry facial expression distractors interfered more than neutral facial expression distractors when judging the sex of target *faces*, this would provide compelling evidence that facial expressions are processed involuntarily, with emotional information from irrelevant angry faces extracted even when targets and distractors are from the *same* stimulus class and even when emotion is completely irrelevant to the required target judgement.

## Methods

### Ethics Statement

Macquarie University's Human Research Ethics Committee approved this research, and all participants gave informed written consent.

### Participants

Thirty participants from Macquarie University were paid $10 for participation. All participants reported normal or corrected-to-normal vision. Data from three participants were excluded as their mean error rates and/or median reaction times were greater than three standard deviations above that of the sample. For the remaining 27 participants (15 male), ages ranged from 18 to 38 years with an average age of 23 years (*SD* = 4.5).

### Stimuli

Six models' faces (three female) were selected from the NimStim Face Stimulus Set (01F, 03F, 10F, 20M, 22M, 23M [26]). An angry and a neutral facial expression were selected from each model, for a total of 12 face images. Twelve lion images were downloaded from the Internet: six lionesses (three angry, three neutral), and six lions (three angry, three neutral). Fourteen participants (six male) who were not involved in the main study were asked to rate the selected face and lion images according to sex (male or female?) and emotion (angry or neutral?) to verify the appropriateness of the selected stimuli. Ratings on both the sex and emotion dimensions exceeded 80% accuracy for both faces and lions, indicating that the sex (male/female) and emotion (angry/neutral) of both the faces and lions could be reliably discriminated.

Black and white line drawings were created from each of the 24 selected images by tracing the images on to transparencies. The transparencies were scanned and two sets of line drawings (one coloured green (RGB = 0, 255, 0) and one coloured red (RGB = 255, 0, 0)) were subsequently produced permitting the images to be superimposed to create composites (see Figure 1).

Composites were created according to four target-distractor pairings: (1) FACE-lion (face target paired with lion distractor); (2) LION-face (lion target paired with face distractor); (3) FACE-face (face target paired with face distractor); (4) LION-lion (lion target paired with lion distractor). Within the produced set of composites, sex congruency (FEMALE-female; FEMALE-male; MALE-female; MALE-male) and emotion congruency (ANGRY-angry; ANGRY-neutral; NEUTRAL-angry; NEUTRAL-neutral) also varied.

For the FACE-lion and LION-face pairings, each of the 12 face images were combined with each of the 12 lion images for a total of 144 composites. Thirty-six of these composites were congruent in terms of both sex and emotion, 36 were sex-congruent but emotion-incongruent, 36 were sex-incongruent but emotion-congruent and 36 were both sex- and emotion-incongruent.

For the FACE-face and LION-lion pairings, each of the 12 face or lion images were combined with 10 face or lion images (as face images of the same identity with different expressions were not paired), for a total of 120 composites. As a result, there were fewer sex-congruent pairings (24 composites each) than sex-incongruent pairings (36 each). To equalise sex- and emotion-congruency proportions, such that 144 composites were shown for each pairing, half of the sex-congruent pairings were shown twice.

### Procedure

Participants were tested individually and were asked to make a sex classification judgement of the targets by pressing labelled keyboard keys (‘M’ for male and ‘F’ for female). For each participant, the target was specified as either the red or green image. The target image was red (and the distractor image was green) for 14 participants and the target image was green for the remaining 13 participants. Participants were asked to respond as quickly and as accurately as possible.

Initially, each of the 24 target images was displayed alone on a white background (in the target colour specified for that participant), and until a response was made, to ensure participants could correctly categorise the sex of these images. Participants correctly categorised each target image twice, before being presented with the composites. In the first practice block of 16 trials, composites were displayed until a response was made. In the second practice block of 16 trials, composites were only displayed for 50 ms. Feedback was provided in these practice blocks and any incorrect trials were repeated until the correct response was provided. The distractor face and distractor lion images used in these practice blocks were not used in the experimental trials.

In the subsequent 576 experimental trials, participants were presented with a fixation cross for 1000 ms followed by a composite for 50 ms, and then a white screen until response. The experiment was controlled by Presentation software (Neurobehavioral Systems Inc.), with trials randomised for each participant.

## Results

Median correct reaction times (RTs) were calculated for all conditions of interest, excluding trials with RTs less than 200 ms.

An initial 4 (target-distractor pairing: FACE-face, FACE-lion, LION-face, LION-lion)×2 (sex congruency: sex congruent, sex incongruent) repeated-measures ANOVA was used to analyse the median RT data. In all analyses, Greenhouse-Geisser correction was applied where the assumption of sphericity was violated.

This initial analysis was performed to ensure that participants were focusing on the sex classification task (i.e., responding to the targets should be quicker when the targets and distractor were congruent in terms of sex than when the targets and distractors were incongruent in terms of sex). This was confirmed through the presence of a significant main effect of sex congruency (*F*(1,26) = 34.30, *p*<.01), with faster responding seen in the sex congruent condition than in the sex incongruent condition (600 ms vs. 619 ms). A significant main effect of target-distractor pairing (*F*(1.71,44.37) = 27.93, *p*<.01), and an interaction between these two variables (*F*(3,78) = 9.89, *p*<.01) were also present. This congruency effect was present for FACE-face and LION-lion target-distractor pairings (both *p*'s<.01), but not for FACE-lion or LION-face pairings (both *p*'s>.05).

Of primary interest, a 4 (target-distractor pairing: FACE-face, FACE-lion, LION-face, LION-lion)×2 (emotion of target: angry/neutral)×2 (emotion of distractor: angry/neutral) repeated measures ANOVA was conducted. This analysis revealed a significant main effect of target-distractor pairing (*F*(1.78,46.32) = 25.87, *p*<.01). While the time taken to classify the sex of the targets in the LION-face pairing was significantly longer than in the LION-lion pairing (*p*<.01), no significant difference in response time emerged for the two target-distractor pairings with face targets (FACE-face and FACE-lion; *p*>.05). This analysis also yielded a significant main effect of emotion of distractor (*F*(1,26) = 15.84, *p*<.01), moderated by a significant interaction between these two variables (*F*(3,78) = 3.78, *p*<.05). No other main effects or interactions were significant.

**Figure 2:**
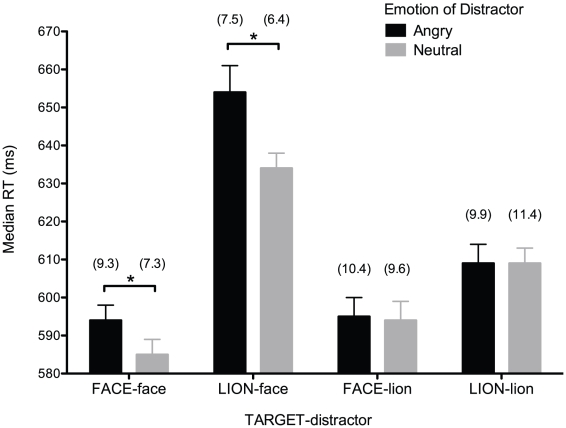
Response times according to target-distractor pairing and emotion of distractor. Means of median RTs (in ms) and percentage error rates (shown in parentheses) for categorising the target image as male or female in each of the target-distractor pairings, shown separately for angry and neutral distractors. Error bars shown reflect the standard error of the means based on within-participant variability (see [27]).

Figure 2 shows that categorising the sex of the target (regardless of the emotion of the target) was slower in the presence of angry than neutral *face* distractors, and this was the case for both face (*p*<.05) and lion (*p*<.01) targets. By contrast, there was no difference between angry and neutral lion distractors in the amount they interfered with categorising the sex of face and lion targets (regardless of the emotion of the target; *p*>.05).

A square root transformation was required to correct for non-normality of the percentage error rate data before the analogous analysis was performed. This analysis showed a significant main effect of target-distractor pairing (*F*(3,78) = 9.44, *p*<.01), again modified by a significant interaction between target-distractor pairing and emotion of distractor (*F*(3,78) = 2.79, *p*<.05) in which more errors were made in processing *face* targets when distractors were angry faces than when they were neutral faces (*p*<.05; see Figure 2).

## Discussion

The current study aimed to test the hypothesis that facial expressions are processed involuntarily., when the emotion of the facial expressions was task irrelevant and the dissimilarity between targets and distractors was minimised.

By asking participants to perform a sex categorisation judgement on the target images, we ensured that the emotional manipulation was entirely unconnected to the demands of the task. The presence of an overall sex congruency effect, averaged across the four target-distractor pairings, verifies that participants were focussing on the sex classification task. Interestingly, the interaction revealed that sex congruency effects were only observed in the FACE-face and LION-lion pairings, indicating that the sex congruency between targets and distractors was only processed for within-category pairings in the current study.

The level of dissimilarity between targets and distractors was reduced from that of previous studies using facial expressions and words by using images of faces and lions as targets and distractors. This resulted in four target-distractor pairings: FACE-face, FACE-lion, LION-face, and LION-lion. Responding to the sex of the target in the LION-face condition was slower overall than in the LION-lion condition, suggesting that faces interfere more than lions with the sex classification task, regardless of the emotion of the distractor images.

For pairings with lion distractors (FACE-lion and LION-lion), we found no evidence to suggest that classifying the sex of targets had been influenced by the emotion of the lion distractor. We suggest that context and experience may play a role in explaining this result: although an angry lion should be a compelling emotional cue from an evolutionary perspective, faces are far more relevant and important in modern human life. The failure to find a difference between angry and neutral lion distractors may also reflect the possibility that neutral and angry lions are equally threatening and interfere with the sex classification task to an equivalent extent.

The pairings with facial expression distractors, however, were the necessary conditions for considering the involuntary nature of facial expression processing. Previous studies have demonstrated that irrelevant facial expression distractors interfere with the classification of superimposed word targets, with such findings suggesting that the facial expressions are being processed involuntarily [5]–[8]. It was anticipated that in the LION-face condition of the current study we would see evidence of irrelevant angry facial expressions interfering with the classification of the superimposed *lion* targets. Results in this condition confirmed this prediction, indicating that response times for classifying the sex of the *lion* targets were increased when presented in conjunction with angry face distractors relative to the neutral face distractors. As this finding is consistent with results from previous studies suggesting involuntary processing of facial expressions, it seems that the processing of task irrelevant facial expressions occurs even when targets are more similar to faces than the previously considered words.

Importantly, results in the FACE-face condition showed more interference from angry face distractors than neutral face distractors with the sex classification of target *faces*. This result indicates that the emotion of an irrelevant distractor face is processed, even when emotion is completely irrelevant to the classification required of the target, and even when that target is another face.

Angry face distractors interfered more than neutral face distractors with responding to both face and lion targets. These results, then, provide clear evidence that emotional facial expressions (at least that of a negative, potentially hostile, expression) are extracted rapidly from a very brief presentation (50 ms) and are involuntarily processed, even when completely task irrelevant. It would be an interesting avenue for future research to consider whether this pattern of results would be modulated by the demands of the task (i.e., explicit emotion categorisation task vs. implicit sex categorisation task).

The presence of interference in the FACE-face condition also suggests that we are able to extract information from an irrelevant angry facial expression distractor at the same time as extracting information from a target face. The results of the current study, then, are consistent with the neuroimaging research suggesting the amygdala responds to emotional information from unattended facial expressions ([15]–[18]; though see [19]).

The importance of facial expressions in our social interactions cannot be overemphasised. Here, we have shown that emotional information from an irrelevant angry facial expression can be processed even when emotion is completely irrelevant to the task, and regardless of the whether the target is a non-face or another face. This provides persuasive evidence for the hypothesis that facial expressions are processed involuntarily.
